# Role of Co-Vapors in Vapor Deposition Polymerization

**DOI:** 10.1038/srep08420

**Published:** 2015-02-12

**Authors:** Ji Eun Lee, Younghee Lee, Ki-Jin Ahn, Jinyoung Huh, Hyeon Woo Shim, Gayathri Sampath, Won Bin Im, Yang–Il Huh, Hyeonseok Yoon

**Affiliations:** 1 School of Polymer Science and Engineering, Chonnam National University, 77 Yongbong-ro, Buk-gu, Gwangju 500-757, South Korea; 2 Department of Polymer Engineering, Graduate School, Chonnam National University, 77 Yongbong-ro, Buk-gu, Gwangju 500-757, South Korea; 3 School of Materials Science and Engineering, Chonnam National University, 77 Yongbong-ro, Buk-gu, Gwangju 500-757, South Korea

## Abstract

Polypyrrole (PPy)/cellulose (PPCL) composite papers were fabricated by vapor phase polymerization. Importantly, the vapor-phase deposition of PPy onto cellulose was assisted by employing different co-vapors namely methanol, ethanol, benzene, water, toluene and hexane, in addition to pyrrole. The resulting PPCL papers possessed high mechanical flexibility, large surface-to-volume ratio, and good redox properties. Their main properties were highly influenced by the nature of the co-vaporized solvent. The morphology and oxidation level of deposited PPy were tuned by employing co-vapors during the polymerization, which in turn led to change in the electrochemical properties of the PPCL papers. When methanol and ethanol were used as co-vapors, the conductivities of PPCL papers were found to have improved five times, which was likely due to the enhanced orientation of PPy chain by the polar co-vapors with high dipole moment. The specific capacitance of PPCL papers obtained using benzene, toluene, water and hexane co-vapors was higher than those of the others, which is attributed to the enlarged effective surface area of the electrode material. The results indicate that the judicious choice and combination of co-vapors in vapor-deposition polymerization (VDP) offers the possibility of tuning the morphological, electrical, and electrochemical properties of deposited conducting polymers.

There is an increasing demand for high-performance electric supply devices with high energy and power density due to the increased social interest in environmentally friendly devices for next-generation energy systems. In representative terms, electrochemical capacitors have strong potential for excellent energy storage characteristics, such as high-efficiency, high-power, high-speed charging, and long life cycle, offering possibilities for creating advanced new techniques in the electronic, automotive, aerospace, and medical industries. Above all, advances in materials synthesis make it possible to devise new devices or to enhance the performance of existing devices.

Conducting polymers have been extensively studied for a variety of electrochemical applications including capacitor electrodes due to their high conductivity, high energy density, and excellent power capability^1^,^2^,^3^. In particular, they have been considered to be key materials for all-organic flexible devices in the future. For a specific application, it is highly important to control the morphology and properties of materials: for example, enlarging the effective surface area of materials is of critical importance for their applications to sensors, filters, membranes, and capacitor electrodes^4^,^5^,^6^,^7^,^8^. In particular, it has been found that the morphological characteristics of conducting polymers even at the microscopic level significantly affect their physical and chemical properties, which can determine the performance of polymer-based devices^9^,^10^,^11^. Unfortunately, however, it is hard to control the morphology of conducting polymers especially at the microscopic scale because of their poor processability. Specifically, they are insoluble in common solvents and are hardly processable even under heat or pressure due to their chain stiffness and strong interchain interaction. Considering these limitations in post-synthesis processability, therefore, it is essential to control the morphology of conducting polymers during the synthesis process.

The vapor-phase polymerization approach has been successfully used to deposit conducting polymers onto substrates, and the influence of vapor-phase over liquid-phase polymerization has been witnessed in the major characteristics of the deposited polymers^12^,^13^,^14^,^15^,^16^. Importantly, vapor deposition polymerization (VDP) can lead to the coating of a polymer layer on any type of substrate regardless of size and shape. In addition, the morphology of the polymer layer can be controlled through judicious choice of synthetic conditions such as temperature and pressure. Also, the significant effect of substrate curvature on the morphology of the deposited polymers was recently demonstrated for the first time by our research group^11^,^13^. Nevertheless, relatively little research has been done on VDP, in contrast with chemical vapor deposition, for fabricating metals or inorganic semiconductors.

Polypyrrole (PPy) is recognized as one of the most promising conducting polymers due to its high conductivity, good environmental stability, biocompatibility, and low cost. Cellulose, a natural polymer, is known to form microfibrils with inherent structural rigidity and high surface area^17^,^18^,^19^. Cellulose as a support has been coupled with functional materials, such as carbon nanotubes and polymers, through various methods^20^,^21^,^22^,^23^,^24^,^25^. Strømme group prepared nanofibrillated cellulose aerogel composites with tunable structural and electrochemical properties^26^, and Ichinose et al achieved uniform PPy coating on individual cellulose fibers by means of polymerization-induced adsorption^27^. Herein, we synthesize PPy/cellulose (PPCL) composite papers, via VDP of pyrrole monomer on cellulose substrate, in the presence of co-vapors. It was anticipated that the co-vapors would participate in the vaporization of monomer, and affect the oxidation level or orientation of polymer chains over polymerization. The PPCL papers were systematically characterized, to provide in-depth insight into how the co-vapors affect the major characteristics of the composite papers. Analytical tools were used for this characterization, such as scanning electron microscopy (SEM), cyclic voltammetry (CV), and galvanostatic charge/discharge measurements. As a result, use of co-vapors in VDP has offered possibility of tuning the physical properties of the deposited polymers as well as the performance of the polymer-based supercapacitors.

## Results and Discussion

### Properties of co-vapors

Table 1 summarizes the main properties of the co-vapors used. The boiling point of pyrrole is higher than those of all the co-vapors used. Therefore, the co-vapors miscible with pyrrole, except water and hexane, play a role in decreasing the boiling point of pyrrole. Dielectric constant is a relative measure of chemical polarity. Difference in polarity allows the classification of co-vapors into two categories, namely, polar (methanol, ethanol, and water), and non-polar vapors (hexane, benzene, and toluene). The polarity of pyrrole is placed in the middle of those of the polar and non-polar vapors. There are further differences in the molecular structure and boiling point of co-vapors. The interaction of pyrrole with co-vapors was estimated by the density functional theory framework at the B3LYP/3-21G level and the results are summarized in Table 2. Pyrrole can have hydrogen bond with methanol, ethanol, and water, in contrast with the non-polar vapors. The dipole moment of pyrrole was calculated to increase when it is coupled with the polar vapors. The increased dipole moment can affect charge transport along the polymer chain as well as chain orientation during polymerization. On the other hand, the non-covalent interaction energy of pyrrole became lower when it is coupled with the non-polar vapors.

### Microstructures of PPCL composite papers

**Figure 1:**
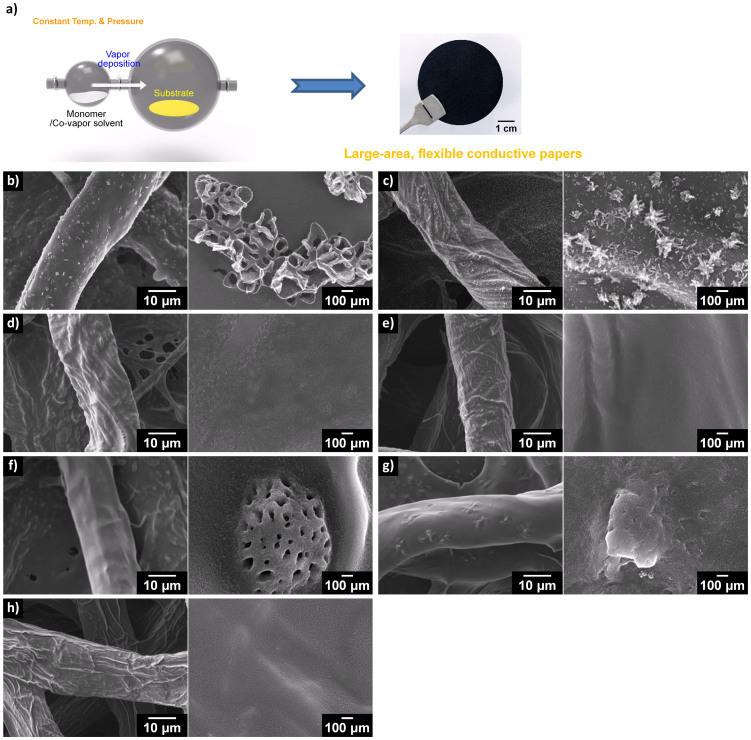
(a) VDP of pyrrole with co-vapors on cellulose substrate and a typical photograph of as-prepared PPCL paper. (b–h) SEM images of PPCL papers prepared with co-vapors: (b) methanol, (c) ethanol, (d) water, (e) hexane, (f) toluene, (g) benzene, and (h) none. (left: low mag., right: high mag.).

Figure 1a briefly describes the overall VDP process of pyrrole monomer with co-vapors on cellulose substrate. The fabrication of PPCL papers involves two processes, when pyrrole and co-vaporized solvent were introduced to ferric chloride-impregnated cellulose substrate in a closed reaction chamber: (i) the diffusion of pyrrole vapor into the cellulose substrate; and (ii) the oxidative polymerization of pyrrole in vapor phase onto the substrate, with ferric chloride as an initiator, at 70°C. The morphologies of the PPCL papers under different co-vapors are compared, and presented in Figure 1b–1h. It is known that low ferric chloride (oxidant) concentration produces thin and uniform polymer layers; while high oxidant concentration yields a thicker and less homogeneous layer of PPy on the cellulose paper. The optimal coating of PPy over cellulose papers was obtained with an oxidant concentration of 2 M, at which condition the large PPCL paper with a diameter of 5 cm (see Figure 1a, the photo) had uniform electrical conductivity over the surface. In SEM images, cellulose paper showed a smooth surface with typical multi-lobe cross-section, and PPy coatings on the cellulose micro-fiber were influenced by the nature of co-vapors introduced together with pyrrole during polymerization. Interestingly, when benzene and toluene were used as co-vapors, small pores were observed over the PPy. Toluene and benzene are well miscible with pyrrole, and the interaction energies of pyrrole-toluene and pyrrole-benzene are lower than that of pyrrole-pyrrole (Table 2). The vapor molecules may strongly interact with pyrrole through π−π stacking interaction over polymerization, and be included in a liquid state inside the PPy, even after the polymerization. The liquid-phase benzene and toluene would be removed during the washing process, resulting in the pores inside the PPy. This explanation is supported by the fact that toluene with a higher boiling point than benzene induced more pronounced porous morphology. Low-boiling-point hexane which is immiscible with pyrrole also gave no significant effect to the surface morphology of PPCL papers. It was also found that the VDP of pyrrole with methanol co-vapor gives rise to porous morphology. The overall structure of the pores generated with methanol was quite different from that of the pores formed with toluene and benzene. Methanol is miscible with pyrrole, and has the lowest boiling point among the co-vapors. It is therefore considered that methanol undergoes vaporization in the polymerization process, leading to a unique porous morphology. The VDP of pyrrole with ethanol co-vapor revealed surface substructures, which were probably due to ferric chloride salts. There were few remarkable characteristics on the surface of PPCL papers prepared with the other co-vapors.

### Polymerization rates

**Figure 2:**
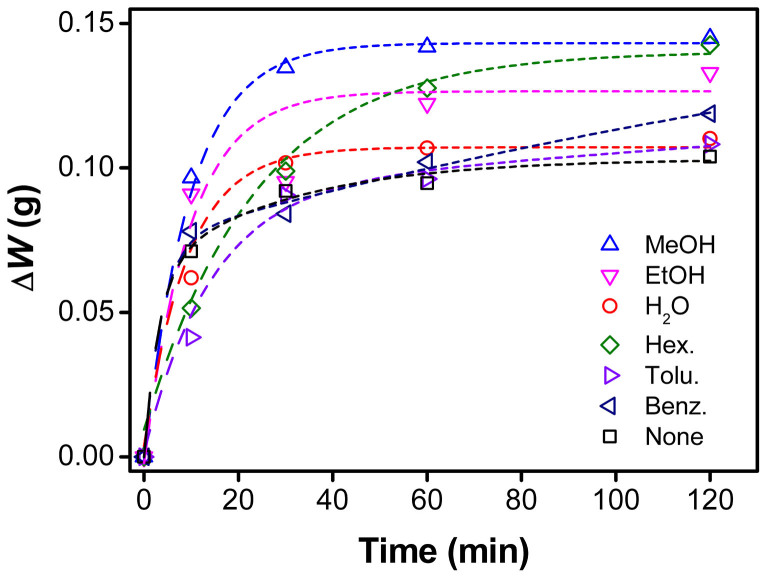
Weight variations showing the amount of the deposited PPy during polymerization.

The weight variation of the deposited PPy was monitored during the VDP to examine how the co-vapor affects the polymerization rate, as plotted in Figure 2. Above all, interestingly, the polymerization yield depended on the boiling point of the co-vapor, regardless of the polarity and miscibility. This result may be associated with the monomer's vapor pressure. In other words, the increased deposition rate would be due to the increased partial pressure of pyrrole in the presence of co-vapor. Additionally, the initial polymerization rate was sensitive to the kind of co-vapors. Polar vapors such as methanol, ethanol, and water appeared to have faster polymerization rates at the initial stage while non-polar vapors such as hexane, toluene, and benzene showed slower polymerization rates. Steric hindrance by the non-polar vapors might be one of the reasons why the initial polymerization rate was retarded.

### Electrical properties

**Figure 3:**
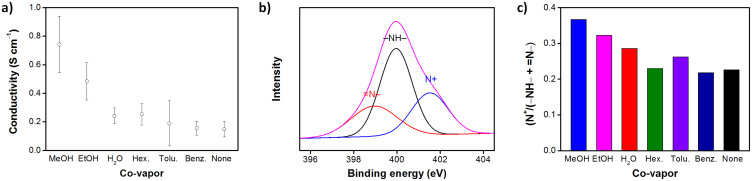
(a) Four-probe conductivities of PPCL papers prepared with different co-vapors. (b) Typical XPS N1s spectrum of PPCL papers and (c) histogram showing the atomic ratios of N^+^ to total N species calculated from the XPS spectra of PPCL papers prepared with co-vapors.

The conductivities of PPCL papers were measured by four probe method and the results are shown in Figure 3a. All PPCL papers prepared with co-vapors showed enhanced conductivities, compared with that of the paper obtained in the absence of co-vapor. The four-probe conductivity of PPCL papers ranged from 0.15 to 0.74 S cm^−1^, which reasonably fits into the conductivity range of only PPy^28^,^29^. This indicates that highly uniform PPy coating was made over the substrate. Notably, methanol and ethanol with high dipole moments of 1.69 and 1.70 D, respectively, had high conductivities, compared to the other co-vapors. It is known that the polar co-vapors can reduce the coulomb interaction between charge carrier and dopant ion, to transiently increase the hopping rate of charge carriers and conductivity^30^. Additionally, the polar co-vapors can enhance the orientation of the polymer chains during polymerization as the secondary dopant^31^, and this seems to allow the enhanced conductivity that persists, even after the removal of co-vapors. Although water has a high dipole moment (1.84 D), it is excluded in the polymerization process, due to its limited immiscibility (solubility of pyrrole in water: 0.68 M) with pyrrole. Consequently, the conductivity of PPCL papers in the presence of water vapor remained almost unchanged.

Quantitative information on the oxidation level of PPCL papers that is proportional to the conductivity was obtained through X-ray photoelectron spectroscopy (XPS) analysis. The imine nitrogen atoms of PPy in the paper are protonated in whole or in part, to yield a range of oxidation states; and thus the oxidation level of the PPCL papers can be quantitatively differentiated, by scrutinizing the XPS spectra of nitrogen atoms around 400 eV. XPS N 1s spectra of PPCL papers prepared with different co-vapors were taken (see Figure S1, Supporting Information) and each spectrum was deconvoluted into three major components, with binding energies at ca. 399.0, 400.2, and 401.5 eV, attributable to quinonoid imine (= N−), benzenoid amine (−NH−), and positively charged nitrogen (N^+^), respectively (Figure 3b)^32^. Positively charged nitrogen species was clearly found in all samples, indicating that the PPy deposited on the cellulose was in an oxidized, namely conductive state. As the oxidation level increases, the intensity of the component corresponding to the positively charged nitrogen increases, along with the decrease in the intensity of the components originating from the quinonoid imine and benzenoid amine. Figure 3c summarizes the atomic ratios of N^+^ to total N species calculated from the XPS spectra. The atomic ratio of N^+^ to total N species was noticeably high for methanol and ethanol vapors, which was consistent with the trend in the conductivity value.

### Electrochemical properties

**Figure 4:**
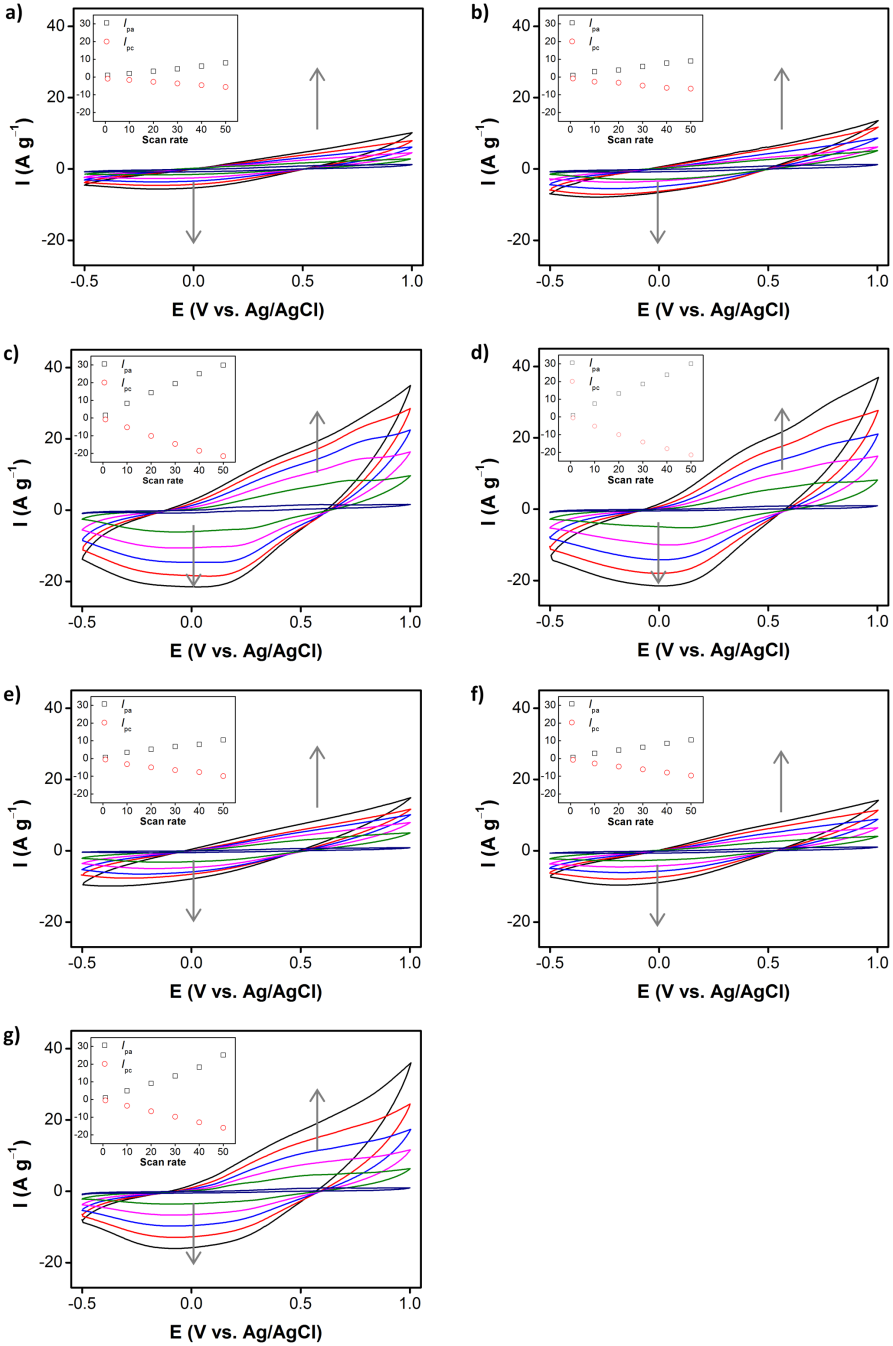
Cyclic voltammograms of PPCL papers prepared with co-vapors: (a) methanol, (b) ethanol, (c) water, (d) hexane, (e) toluene, (f) benzene, and (g) none. Insets: Plots of the peak current (the anodic peak current, Ipa; the cathodic peak current, Ipc) vs. the scan rate extracted from the cyclic voltammograms.

**Figure 5:**
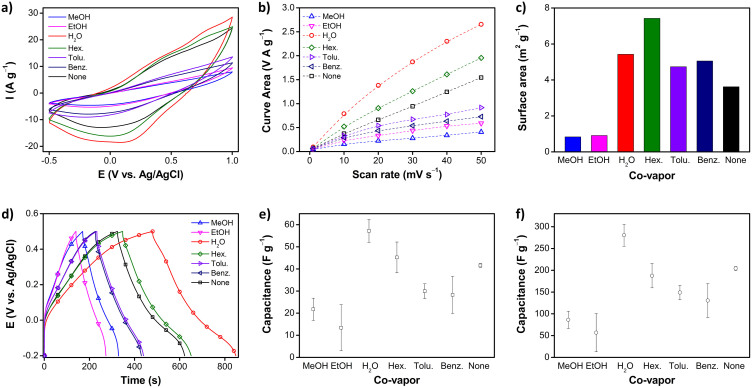
(a) Cyclic voltammograms of PPCL papers prepared with various co-vapors at a scan rate of 40 mV s^−1^, and (b) their integrated areas. (c) BET surface areas of PPCL papers prepared with different co-vapors (the BET surface area of only cellulose paper was 12.5 m^2^ g^−1^). (d) Charge/discharge curves, and (e) specific discharge capacitances, measured at a current density of 0.1 A g^−1^. (f) Specific discharge capacitances calculated using the amount of only deposited PPy in (e).

The electroactivity of PPCL papers was examined, using CV analysis. Figure 4 exhibits CV curves of PPCL papers recorded at different scan rates in the potential range of −0.5 to 1.0 V, using 1 M sulfuric acid electrolyte solution. The CV curves all had a similar shape in the corresponding potential range. A pair of broad peaks originating from the redox switch of PPy was observed. The effect of the potential scan rate on the peak current was monitored in the range of 1–50 m V s^−1^. Figure 4 inset shows the relationship between the peak current and the scan rate in the CV curves. Both anodic and cathodic peak currents increased linearly with scan rate, indicating that the PPCL electrode kinetics are subject to a surface-controlled redox process. Namely, PPy thin layer would be deposited on cellulose substrate, and thus the redox process was likely to be confined to the surface of the PPCL electrode. In general, the CV curve enlarges, as the scan rate increases. Additionally, the area of the CV curves was found to be dependent on the type of co-vapor used. Representatively, the CV curves of PPCL papers at a scan rate of 40 mV s^−1^ are compared (Figure 5a), and the calculated CV curve areas are plotted (Figure 5b). The CV curve area is indicative of the surface area and capacitance of the electrode material. The integrated areas of the CV curves at the same scan rate clearly increased, in the order of methanol < ethanol < benzene < toluene < none < hexane < water. The BET surface area was supplementarily measured, to gain rough information of the surface area of the specimens. Figure 5c presents a histogram showing the surface area of PPCL papers prepared with different co-vapors. There was a difference in the trend observed from the CV curve area, because the BET surface area can include the area of cellulose substrate, as well as PPy. However, it is evident that immiscible co-vapors such as water and hexane aid in the yielding of higher electroactive surface areas of PPy. Higher surface area commonly stems from two reasons: i) porous structure and ii) smaller or thinner dimensions. There were no remarkable porous structures in the PPCL paper prepared with water and hexane; and thus it is believed that an immiscible co-vapor facilitates the formation of uniform, thinner PPy layer on the cellulose during VDP.

### Capacitive behavior

It is known that PPy stores electrical charges, through a pseudocapacitive charge storage mechanism mediated by redox reaction. Pseudocapacitance stems from reversible surface or near-surface reactions for charge storage^33^. The specific capacitances of PPCL papers were measured at a current density of 0.1 A g^−1^ in acidic aqueous electrolyte, and the results are presented in Figure 5d and 5e for comparison. The specific capacitance of PPCL papers was sensitive to which co-vapor was used during the VDP. Water co-vapor renders the highest capacitance of 57.2 F g^−1^, while ethanol co-vapor gave the lowest capacitance of 13.4 F g^−1^. The specific capacitance increased in the order of ethanol < methanol < benzene < toluene < none < hexane < water. Such trend was almost in accordance with that observed in the CV curve area, except for ethanol and methanol. Methanol co-vapor induced higher conductivity of PPCL, than ethanol co-vapor. Additionally, it was found in the CV analysis that the redox reaction of PPCL paper electrodes occurred at the PPy surface. Considering these results, under our experimental conditions, two factors of surface area and conductivity of PPCL papers affect their specific capacitance, and in particular, the surface area is more critical than the conductivity, in determining the specific capacitance. Figure 5f shows the specific capacitance calculated using the amount of only deposited PPy, where the capacitance increased to the max. 280 F g^−1^.

### Mixed co-vapors

**Figure 6:**
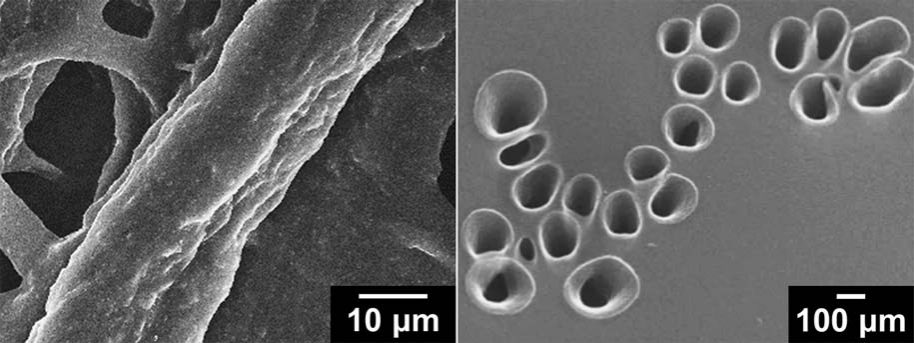
SEM images of PPCL papers prepared with both methanol and water co-vapors (left: low mag., right: high mag.).

**Figure 7:**
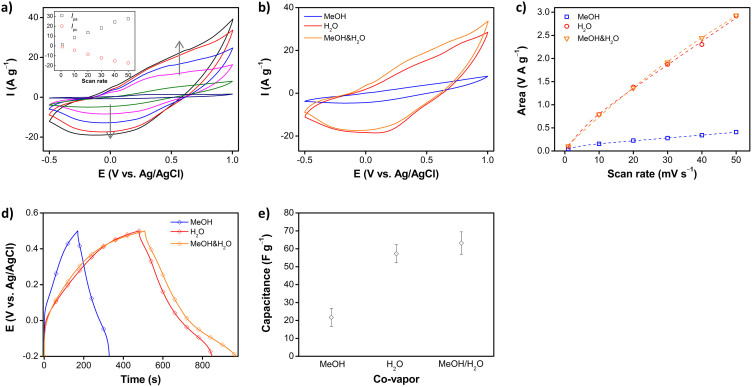
(a) Cyclic voltammograms of PPCL papers prepared with both methanol and water co-vapors (Insets: Plots of the peak current). (b) Representative cyclic voltammograms of PPCL papers prepared with single co-vapor (methanol or water) and mixed co-vapors (methanol and water) at a scan rate of 40 mV s^−1^, and (c) the calculated curve areas therefrom. (d) Representative charge/discharge curves of PPCL papers prepared with single co-vapor (methanol or water) and mixed co-vapors (methanol and water), at a current density of 0.1 A g^−1^, and (e) the calculated specific capacitances therefrom.

The electrical conductivity and electroactivity of PPCL papers were found to be positively affected by methanol and water co-vapors, respectively. Therefore, it was further examined how the properties of PPCL papers become different, in the presence of the two co-vapors during VDP. The molar ratio of pyrrole:methanol:water used was 1:1:1. First, Figure 6 displays SEM image of PPCL papers prepared with both methanol and water co-vapors. Unique surface pores were observed, which was similar to the surface morphology of PPCL papers prepared with only methanol co-vapor. The conductivity was measured to be 1.78 ± 0.2 S cm^−1^, even 2.4 times higher than that of PPCL papers obtained with methanol co-vapor. Namely, it is evident that there was a positive synergistic effect on the properties of PPCL papers, when the two co-vapors were used. Figure 7a shows CV curves recorded at different scan rates. Compared to other PPCL papers, there was no noticeable difference in the CV curve shape. Representative CV curves and the calculated curve areas are presented in Figures 7b and 7c for comparison. The curve area was found to be comparable to that of PPCL papers prepared with only water co-vapor. Similarly, the charge/discharge behavior was examined. As seen in Figure 7d and 7e, it turned out that using the two co-vapors produces ca. 10% improved specific capacitance as compared to only water co-vapor. These results support the thesis that by judicious choice and combination of the co-vapors in VDP, the morphological, electrical, and electrochemical properties of vapor-phase deposited PPy can be tuned.

## Conclusions

The role of co-vapors in VDP was examined, using PPy conducting polymer and cellulose substrate as a model example. Co-vapors would affect the i) vaporization of pyrrole, ii) orientation of forming PPy chains, iii) rate and efficiency of oxidative polymerization, iv) swelling of the substrate, and v) diffusion of pyrrole into the substrate during VDP, all of which lead to meaningful changes in the major characteristics of PPCL composite papers. Under our experimental conditions, unique surface porous structures were generated in the presence of benzene and toluene co-vapors, and relatively thin and uniform PPy coating on cellulose can be made with the aid of water and hexane. Using alcohol co-vapors leads to higher electrical conductivities in PPy. PPCL papers hold great potential for practical applications in various areas, such as sensors, catalysis, and energy^33^,^34^,^35^,^36^,^37^. It is believed that understanding such mechanisms may provide an efficient route to tuning the properties of conducting polymer composites, for specific applications.

## Methods

### Materials

Pyrrole (98%) was purchased from Sigma-Aldrich, and ferric chloride (FeCl_3_) (≥98%) was obtained from Merck. Alpha-cellulose paper (>98%) as a substrate was obtained from Whatman. Nickel plate was used as a working electrode, and carbon paste was employed as a binder. All solvents were used as received, and distilled water was used for all experiments.

### Fabrication of PPCL composite papers

PPCL composite papers with a diameter of 5 cm were obtained via VDP. Cellulose papers were impregnated with 2 M ferric chloride solution (0.4 mL) and then allowed to dry in vacuum oven at 25°C for 24 h. The cellulose paper was placed into a closed reaction chamber, and then an amount of pyrrole monomer was introduced into the chamber with co-vapors, in 1:1 molar ratio. The polymerization proceeded for 2 h at 70°C, at atmospheric pressure. Subsequently, the resulting PPCL papers were washed with excess distilled water to remove residual impurities, and then dried in vacuum oven for 12 h. Methanol, ethanol, water, benzene, hexane, and toluene were used as the co-vapor.

### Characterization

The morphological characteristics of PPCL papers were analyzed using JEOL JSM-7500F scanning electron microscopy. Specimens for SEM were coated with a thin layer of gold. The electrical conductivities were measured at room temperature by standard four-probe method. XPS was performed, by Thermo VG Scientific Multilab 2000 spectrometer with an Mg/Al twin-anode excitation source. Peak fitting of the collected spectra was conducted with VG Avantage software. CV and charge/discharge characteristics were examined, using a three-electrode cell containing 1 M H_2_SO_4_ solution, with Pt counter electrode, and Ag/AgCl reference electrode. As a working electrode, PPCL papers of 0.5 cm × 1.5 cm dimensions were attached to a nickel plate. All electrochemical measurements were conducted using Metrohm Autolab B.V. PGSTAT101 potentiostat/galvanostat. The BET surface area and pore volume were calculated, using Fisons Instruments, Sorptomatic 1990.

## Supplementary Material

Supplementary Information Supplementary Information

## Figures and Tables

**Table 1:** Main properties of pyrrole and co-vapors

Co-vapor	Boiling point (°C)	Dielectric constant	Dipole moment (D)	Miscibility with pyrrole
Pyrrole	130.0	7.50	1.80	
Methanol	65.0	33.00	1.70	Miscible
Ethanol	79.0	24.60	1.69	Miscible
Water	100.0	80.00	1.84	Immiscible*
Hexane	68.7	1.88	0.08	Immiscible
Toluene	111.0	2.38	0.36	Miscible
Benzene	80.0	2.30	0.00	Miscible

*Pyrrole is soluble in water only at a small amount of 60 g L^−1^.

**Table 2:** Calculated interactions between pyrrole and co-vapors*

Pyrrole/Co-vapor	3D graphic**	Energy (a.u.)	Dipole moment (D)	H-bonding
Pyrrole/Pyrrole	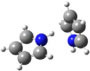	−418.0	3.25	None
Pyrrole/Methanol	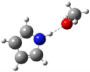	−324.1	5.35	Possible
Pyrrole/Ethanol	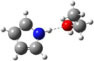	−363.2	4.80	Possible
Pyrrole/Water	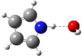	−285.0	5.09	Possible
Pyrrole/Hexane	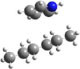	−444.8	1.94	None
Pyrrole/Toluene	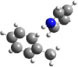	−479.1	2.44	None
Pyrrole/Benzene	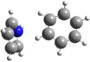	−440.0	2.02	None

*All calculations were carried out using the Gaussian 09 program suite.

**The dotted line indicates hydrogen bond between the corresponding atoms.
