# Supplementary material for: Role of Co-Vapors in Vapor Deposition Polymerization
Source: Sci Rep. 2015 Feb 12;5:8420. doi: 10.1038/srep08420 (PMC5389134; doi:10.1038/srep08420)
Supplement: Supplementary Information [file srep08420-s1.pdf]

Supporting Information

Role of Co-Vapors in Vapor Deposition Polymerization

Ji Eun Lee,[c] Younghee Lee,[c] Ki-Jin Ahn,[c] Jinyoung Huh,[c] Hyeon Woo Shim,[c] Gayathri Sampath,[c] Won Bin Im,[d] Yang-Il Huh,[b,c] and Hyeonseok Yoon[a,b,c,*]

[a]Alan G. MacDiarmid Energy Research Institute, [b]School of Polymer Science and Engineering, Chonnam National University, 77 Yongbong-ro, Buk-gu, Gwangju 500-757, South Korea.
[c]Department of Polymer Engineering, Graduate School, Chonnam National University, 77 Yongbong-ro, Buk-gu, Gwangju 500-757, South Korea.
[d]School of Materials Science and Engineering, Chonnam National University, 77 Yongbong-ro, Buk-gu, Gwangju 500-757, South Korea.

*Address correspondence to hyoon@chonnam.ac.kr

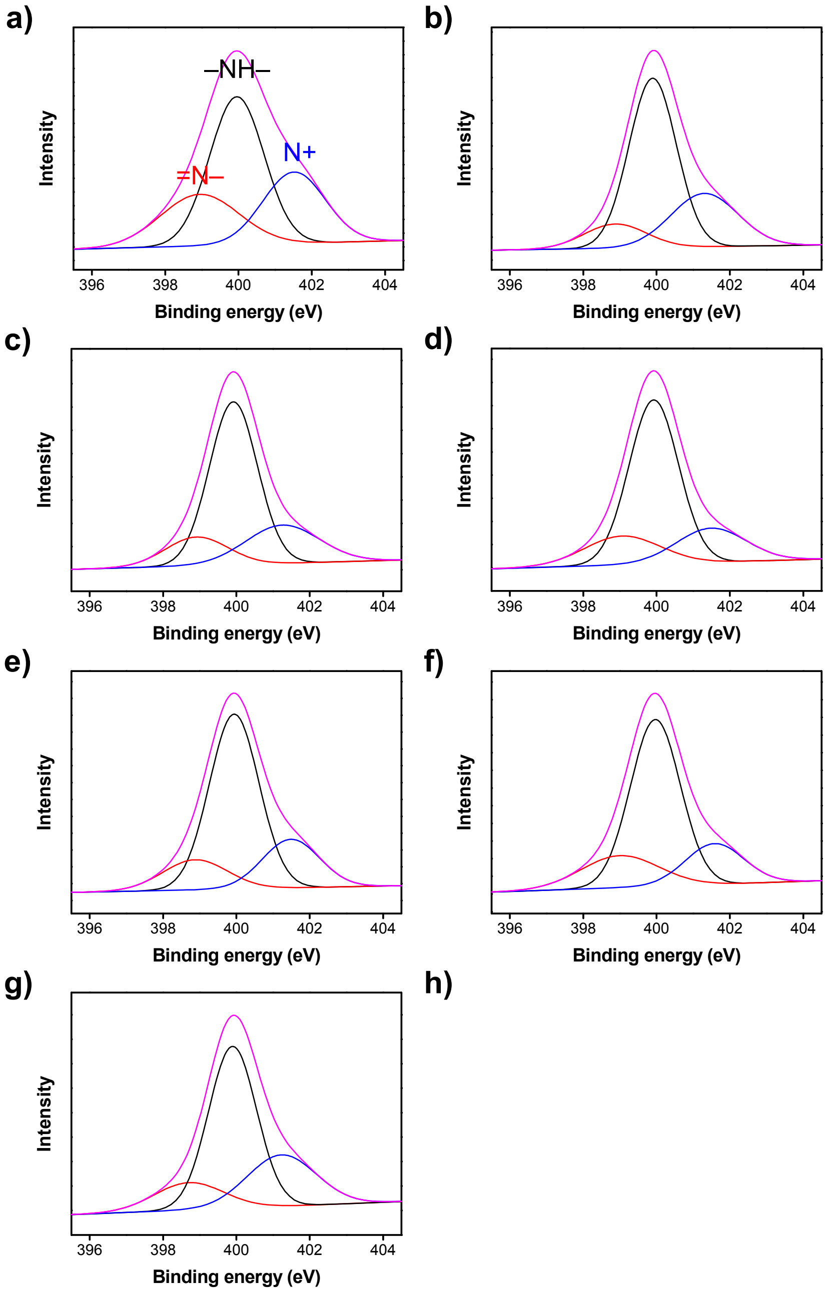

Figure S1. XPS N1s spectra of PPCL papers prepared with different co-vapors: (a) methanol, (b) ethanol, (c) water, (d) hexane, (e) toluene, (f) benzene, and (g) none.
